# Membrane skeleton hyperstability due to a novel alternatively spliced 4.1R can account for ellipsoidal camelid red cells with decreased deformability

**DOI:** 10.1016/j.jbc.2023.102877

**Published:** 2023-01-06

**Authors:** Yuqi Chen, Kosuke Miyazono, Yayoi Otsuka, Mariko Kanamori, Aozora Yamashita, Nobuto Arashiki, Takehisa Matsumoto, Kensuke Takada, Kota Sato, Narla Mohandas, Mutsumi Inaba

**Affiliations:** 1 Laboratory of Molecular Medicine, Graduate School of Veterinary Medicine, Hokkaido University, Sapporo, Japan; 2 Department of Biochemistry, School of Medicine, Tokyo Women’s Medical University, Tokyo, Japan; 3 Drug Discovery Structural Biology Platform Unit, RIKEN Center for Biosystems Dynamics Research, Yokohama, Japan; 4 Red Cell Physiology Laboratory, New York Blood Center, New York, New York, USA

**Keywords:** alternative splicing, camelid, elliptocyte, erythrocyte, hereditary elliptocytosis, membrane protein, membrane structure, plasma membrane, protein 4.1R, spectrin, GST, glutathione-S-transferase, HE, hereditary elliptocytosis, MCHC, mean corpuscular hemoglobin concentration, RBC, red blood cell, SABD, spectrin–actin-binding domain

## Abstract

The red blood cells (RBCs) of vertebrates have evolved into two basic shapes, with nucleated nonmammalian RBCs having a biconvex ellipsoidal shape and anuclear mammalian RBCs having a biconcave disk shape. In contrast, camelid RBCs are flat ellipsoids with reduced membrane deformability, suggesting altered membrane skeletal organization. However, the mechanisms responsible for their elliptocytic shape and reduced deformability have not been determined. We here showed that in alpaca RBCs, protein 4.1R, a major component of the membrane skeleton, contains an alternatively spliced exon 14–derived cassette (e14) not observed in the highly conserved 80 kDa 4.1R of other highly deformable biconcave mammalian RBCs. The inclusion of this exon, along with the preceding unordered proline- and glutamic acid–rich peptide (PE), results in a larger and unique 90 kDa camelid 4.1R. Human 4.1R containing e14 and PE, but not PE alone, showed markedly increased ability to form a spectrin–actin–4.1R ternary complex in viscosity assays. A similar facilitated ternary complex was formed by human 4.1R possessing a duplication of the spectrin–actin-binding domain, one of the mutations known to cause human hereditary elliptocytosis. The e14- and PE-containing mutant also exhibited an increased binding affinity to β-spectrin compared with WT 4.1R. Taken together, these findings indicate that 4.1R protein with the e14 cassette results in the formation and maintenance of a hyperstable membrane skeleton, resulting in rigid red ellipsoidal cells in camelid species, and suggest that membrane structure is evolutionarily regulated by alternative splicing of exons in the 4.1R gene.

Although cell shape and function are intrinsically coupled, the shapes of red blood cells (RBCs) in vertebrate species are highly diverse, despite these cells sharing a common function in efficient gas exchange in all vertebrates. Vertebrate RBCs have evolved into two basic shapes. All nonmammalian RBCs have a biconvex ellipsoidal shape and are nucleated (1, 2). The generation and maintenance of this elliptical shape likely result from cooperative and anisotropic interactions of the plasma membrane–associated membrane skeleton and the transcellular cytoskeleton, involving both intermediate filaments and microtubules (1). In contrast, due to the absence of a transcellular cytoskeleton, nucleus, and intracellular organelles, the shape of mammalian RBCs depends exclusively on their plasma membranes, with uniform membrane skeletal structure across the entire surface (3). The resultant RBCs of most mammalian species have a biconcave disk shape under hydrostatic conditions and undergo extensive and repeated deformation under shear stress during transit through the microvasculature, resulting in efficient gas and ion transport (4, 5, 6). However, the evolutionary molecular mechanism responsible for the difference between ellipsoidal- and discoidal-shaped RBCs remains unclear.

Camelid RBCs differ in shape from other RBCs. In contrast to other mammalian species, mature RBCs (erythrocytes) of camelid species are flat ellipsoids with markedly reduced membrane deformability in response to applied shear stress (7). Because polychromatophilic RBCs are round, camelid RBCs likely acquire their ellipsoid shape during maturation of reticulocytes into erythrocytes (8), with a circumferential bundle of microtubules, called the marginal band, likely being involved in this transformation of camelid RBCs (9). Marginal bands, however, are not required for the maintenance of ellipticity after the cells have fully matured; thus, this filamentous structure is present in reticulocytes but absent in erythrocytes (9). The marginal band microtubules, characteristic of all nucleated nonmammalian RBCs (1), have also been implicated in the formation of flat discoid–shaped avian erythrocytes, with these cells subsequently acquiring an elliptic shape (10, 11). These findings suggest that the microtubules and membrane skeleton have different roles in flat ellipsoid shape formation and that the membrane skeleton provides the primary molecular basis for the ellipsoid shapes of both camelid and nonmammalian RBCs. The characterization of membrane skeletons in camelid RBCs may therefore enable a determination of the mechanisms that underlie the evolutionary divergence of RBC shapes.

The membrane skeleton is composed of rod-like (αβ)_2_ spectrin tetramers, which are interconnected in a uniform hexagonal lattice through binding to short F-actin at the junctional complex, a process facilitated and stabilized by the protein 4.1R (5, 12, 13). Protein 4.1R binds to both spectrin and F-actin through the 10 kDa spectrin–actin-binding domain (SABD), which in turn is associated with the transmembrane proteins, glycophorin C and band 3, through the N-terminal membrane-binding domain (also designated the FERM domain), connecting the membrane skeleton to the lipid bilayer (12, 14). The expression of 4.1R is spatiotemporally regulated by alternative splicing and differential usage of two distinct initiation codons, resulting in the expression of multiple isoforms during terminal erythroid differentiation (15, 16). In late-stage erythroblasts and reticulocytes, the expression of an alternative exon 16, encoding the N-terminal 21-amino acid (aa) cassette in the SABD, results in the synthesis of the predominant 80 kDa isoform of 4.1R (4.1R^80^) (17), consisting of a doublet of the polypeptides 80 kDa 4.1Ra and 78 kDa 4.1Rb, in mature RBCs of most mammals (18, 19). This critical 21-aa cassette is necessary for high-affinity binding to spectrin and actin at the junctional complex and stabilization of the RBC membrane skeleton (20, 21). Thus, the membrane-associated two-dimensional skeletal network maintains the mechanical stability and integrity of RBCs (4, 5, 6). Although the features of several membrane proteins, including spectrin, band 3, and 4.1R, have long been regarded as associated with the elliptic shape of camelid RBCs (22, 23, 24, 25), the molecular mechanisms associated with the unique morphological features of camelid RBCs and the altered mechanical properties of their membranes remain unclear.

The structural defects of membrane skeletal proteins that weaken or disrupt membrane integrity appear to facilitate a shear stress-induced rearrangement of membrane skeletons, precluding recovery of the biconcave shape and lead to the formation of permanently deformed RBCs, called elliptocytes (4, 5, 26, 27). Various genetic defects of spectrin and 4.1R have been reported to cause hereditary elliptocytosis (HE), in which the degree of hemolytic anemia is dependent on the severity of reduction in the lateral linkages in the membrane skeleton (4). Structural defects of 4.1R are much less common than spectrin mutations in HE pathogenesis. However, some 4.1R mutations, including deletion mutations in the SABD associated with moderate anemia (17, 28, 29) and intramolecular duplication of SABD associated with a mild phenotype (30, 31, 32), have defined the functional significance of the SABD. Camelid 4.1R proteins appear to have a molecular mass higher than the highly conserved 4.1R^80^ (22, 25). It is of interest to determine whether, as in asymptomatic HE due to SABD duplication with a 95 kDa 4.1R mutant (4.1R^95^) (30, 32), camelid 4.1R has features distinct from other mammalian 4.1R proteins, resulting in the rearrangement of membrane skeletal organization following exposure to shear stress in the circulation and the consequent elliptocytic shape of camelid RBCs.

The objective of the present study was to explore the molecular basis for elliptocytic RBC formation in camelids, focusing on the unique 4.1R isoform with a molecular mass of 90 kDa (4.1R^90^). We first analyzed mRNA from alpaca reticulocytes and examined the amino acid sequence of 4.1R^90^ isolated from alpaca RBC membranes. The data showed that alpaca 4.1R^90^ contained an additional 19-aa cassette encoded by alternate exon 14 adjacent to the N-terminal of SABD that is not included in other mammalian 4.1R^80^ proteins. A unique amino acid sequence derived from the downstream region of exon 13 was also found in 4.1R^90^. Several recombinants of human 4.1R^80^–containing alpaca sequences were used to study their ability to form spectrin–actin–4.1R ternary complex (13) by measuring the viscosity of the complexes and also to examine the ability to interact with β-spectrin (33). The results showed that the exon 14–derived 19-aa cassette (e14) in alpaca 4.1R^90^, as well as the duplicated SABDs in human 4.1R^95^ mutant (30, 32), markedly enhanced ternary complex formation. The findings of this study suggest that an unusual sequence adjacent to the SABD causes a hyperstabilization of the membrane skeleton with reduced membrane plasticity, leading to the formation of elliptocytic RBCs in camelid species.

## Results

### Identification of 4.1R in alpaca RBC membranes

**Figure 1:**
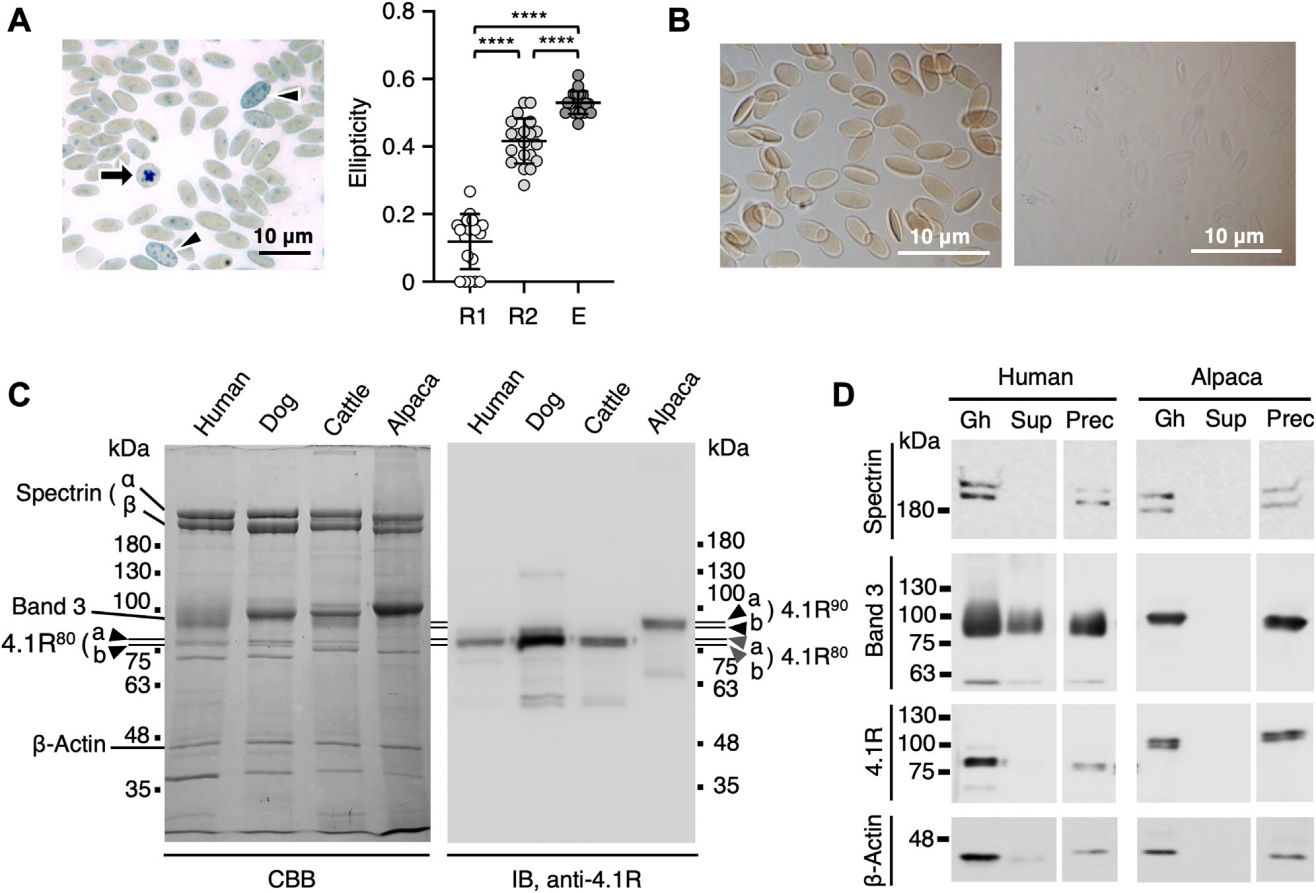
**Characterization of the ellipticity of alpaca RBCs and the 90 kDa 4.1R in alpaca RBC membranes.***A*, *Left panel*. A new methylene *blue*–stained smear of freshly obtained alpaca peripheral blood, showing a younger aggregate reticulocyte (*arrow*) and punctate reticulocytes (*arrowhead*). *Right panel*. Ellipticity of aggregate (*R1*) and punctate (*R2*) reticulocytes and erythrocytes (*E*). Data are expressed as the mean ± S.D. (n = 20). ∗∗∗∗*p* < 0.0001 by one-way ANOVA with Tukey’s multiple comparison test. Bar represents 10 μm. *B*, alpaca RBCs washed in PBS (*left*) and ellipsoid Triton shell after solubilization of membranes in PBS containing 1% Triton X-100 on ice for 30 min (*right*). Bars, 10 μm. *C*, 8% SDS-PAGE gel of membrane proteins from human, canine, bovine, and alpaca RBCs stained with Coomassie brilliant *blue* (*CBB*, 7 μg/lane) and immunoblotted with anti-canine 4.1R antibody (*IB*, 3 μg/lane). The migration of alpaca 4.1R (*4.1R*^*90*^, *a* and *b*) was slower than that of human, canine, and bovine 4.1R proteins (*4.1R*^*80*^, *a* and *b*). *D*, membrane ghosts (*Gh*) from human and alpaca RBCs were incubated in 1% Triton X-100 solution on ice for 1 h followed by centrifugation at 100,000*g* for 30 min to separate the supernatant (*Sup*) and precipitate (*Prec*). Samples equivalent to ghosts containing 2 μg of proteins were subjected to SDS-PAGE and immunoblotting to detect spectrin (*Spectrin*), band 3 (*Band 3*), 4.1R (*4.1R*), and β-actin (*β-Actin*). The migrating positions of size markers are shown in kDa. RBC, red blood cell.

The ellipticity of alpaca erythroid cells increased during terminal erythroid differentiation as immature reticulocytes matured into erythrocytes (Fig. 1*A*), indicating that ellipsoid shape formation is triggered by the mechanical stress-induced membrane structural organization in the circulation (8). Alpaca RBCs retained their ellipsoid shape following solubilization of the plasma membrane in 1% Triton X-100, suggesting that membrane skeletal organization was irreversibly altered during the formation of elliptic RBCs (Fig. 1*B*).

As in human RBCs, the predominant component of the major protein 4.1R in the membranes of most mammalian RBCs consists of a doublet of polypeptides (4.1R^80^) (18). Immunoblotting analysis showed that the predominant form of 4.1R in alpaca RBC membranes was a doublet of 90 kDa and 88 kDa polypeptides (4.1R^90^), with a size apparently larger than that of 4.1R^80^ in other mammalian RBCs (Fig. 1*C*). A similar 4.1R^90^ protein was detected in camel RBC membranes (Fig. S1); results consistent with previous findings indicate that the 4.1R protein in camelid RBCs had a higher molecular mass on SDS-PAGE than other mammalian 4.1R proteins (22, 25). Following solubilization of membranes with Triton X-100, alpaca 4.1R^90^ was detected in the insoluble precipitate (Triton shell), along with other membrane skeletal proteins including spectrin and actin (Fig. 1*D*), indicating that 4.1R^90^ participates in the formation and stabilization of the membrane skeleton in alpaca RBCs. Band 3 was not detected in the supernatants of alpaca RBC membranes, consistent with findings on llama RBC membranes (24), suggesting that most band 3 is bound to the membrane skeleton in alpaca RBCs. Although the relative abundance of band 3 was higher in alpaca than human RBC membranes (Fig. 1*C*) (23, 24), immunoblotting showed no significant difference in signal intensity between alpaca and human band 3 (Fig. 1*D*). However, the anti-band 3 antibody used in the present study was raised against band 3 polypeptides purified from dog RBC membranes (34) and had greater cross-reactivity with human than bovine and alpaca band 3 protein.

### Alpaca 4.1R^90^ contains characteristic amino acid sequences derived from exons 13 and 14

**Figure 2:**
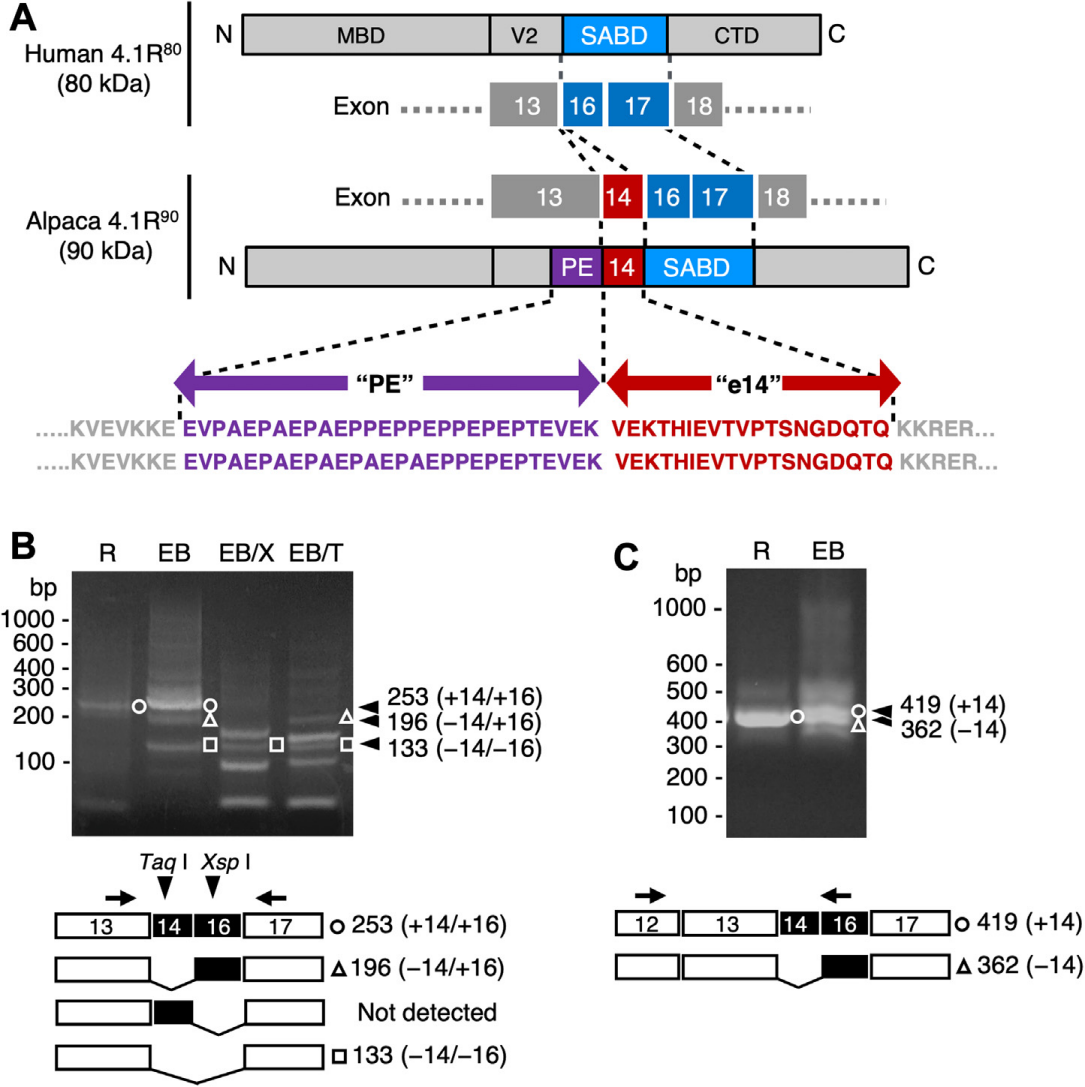
**Involvement of the exon 14–derived sequence in alpaca 4.1R**^**90**^**cDNA.***A*, cDNA and amino acid sequence analyses of alpaca 4.1R^90^, focusing on the regions derived from exons 13–17, in comparison with the relevant structures of human 4.1R^80^. Structural domains of 4.1R^80^ were assigned as described (35): CTD, C-terminal domain; MBD, membrane-binding domain; SABD, spectrin–actin-binding domain; V2, variable region 2. The C-terminal region of V2, which is encoded by exon 13, of alpaca 4.1R^90^ contains a unique amino acid sequence “PE” (shown in *purple letters*) with two allelic differences. The exon 14–derived sequence “e14” is flanked by the PE and SABD sequences and is shown in *red*. The complete amino acid sequence and an example of LC-MS/MS analysis of alpaca 4.1R^90^ are shown in the Supporting Information (Fig. S2 and Table S1). *B*, cDNA fragments encompassing exons 13–17 that were PCR amplified from alpaca reticulocyte (*R*) and day 6 erythroblasts (*EB*) using the primers Vic41.5′e13PE.F and Vic41.e17.KKHHASI.R. The arrows show the positions of the primers. The cDNA fragments obtained include a 253 bp fragment containing nucleotides derived from exons 14 and 16 (*○, +14/+16*), a 196 bp fragment with an exon 16 sequence (*△, −14/+16*), and a 133 bp fragment lacking sequences from exons 14 and 16 (*□, −14/−16*). Because exons 14 and 16 contain unique restriction sites for *Taq I* and *Xsp* I, respectively (*arrowheads*), digestion with *Xsp* I (*EB/X*) or *Taq* I (*EB/T*) confirmed that the 253 bp and 196 bp fragments contain exon 14–derived sequence, whereas only 253 bp fragment contains exon 16–derived nucleotides. A cDNA fragment containing exon 14–derived nucleotides alone was not detected. *C*, cDNA fragments encompassing exons 12–16 PCR amplified from reticulocyte (*R*) and erythroblasts (*EB*) using the primers Vic41.e12.TRQASA.F and Vic41.seq.e16.R2 (*arrows*). The 419 bp fragment containing the exon 14 sequence (*○, +14*) and the 362 bp fragment without this sequence (*△, −14*) are indicated. SABD, spectrin–actin-binding domain.

Based on the 4.1R sequences in the alpaca genomic DNA sequence database (VicPac3.1), alpaca 4.1^90^ cDNAs prepared from mRNAs of reticulocytes in peripheral blood were cloned and analyzed. The resultant complementary DNA (cDNA) consisted of exons 4–21, except for exon 15 (Fig. S2). The predicted amino acid sequence of alpaca 4.1^90^ consisted of 652 amino acid residues, 30 amino acid residues larger than human 4.1R^80^, and showed high similarity to the sequence of human 4.1R^80^. The predicted alpaca 4.1R^90^ sequence contained two peptides adjacent to the SABD but not found in human 4.1R^80^. The first sequence derived from the region downstream of exon 13 was located at the C-terminus of the V2 region (35) flanked by the membrane-binding domain and SABD and contained many proline (Pro) and glutamic acid (Glu) residues (Figs. 2*A* and S2). This Pro- and Glu-rich region, designated the PE region, also showed genotypic variation, with one variation containing three Pro-Ala-Glu and three Pro-Pro-Glu repeats and the other containing five Pro-Ala-Glu repeats and one Pro-Pro-Glu sequence.

The other sequence characteristic of alpaca 4.1R^90^ was encoded by exon 14, designated e14; this 19-aa peptide was flanked by the PE sequence and the SABD. The amino acid sequence of e14, as well as that of the SABD, was found to be identical or highly conserved among mammalian species (Fig. S3). Amplification of cDNAs encompassing exons 1317 from alpaca reticulocytes and day 6 erythroblasts generated three distinct fragments, which contained sequences from both exons 14 and 16 (Fig. 2*B*, *+14/+16*), exon 16 alone (*−14/+16*), or none of these exons (*−14/−16*). The +14/+16 fragment was predominant in both reticulocyte and erythroblast PCR products, with relative abundances of ∼75% and ∼42%, respectively. No fragment possessing the sequence from exon 14 but not from 16 was detected. Moreover, PCR amplification of exons 12–16 generated only a 419 bp fragment containing the exon 14–derived sequence from reticulocyte cDNAs (Fig. 2*C*, *+14*), whereas nearly equivalent amounts of two distinct products, one with (*+14*) and the other without (*−14*) the exon 14–derived sequence, were obtained from erythroblast cDNAs (Fig. 2*C*). These data demonstrate that exons 14 and 16 are alternatively spliced in alpaca erythroid cells and that a combination of these exons is expressed in the major 4.1R mRNA species in alpaca erythroblasts and reticulocytes.

**Figure 3:**
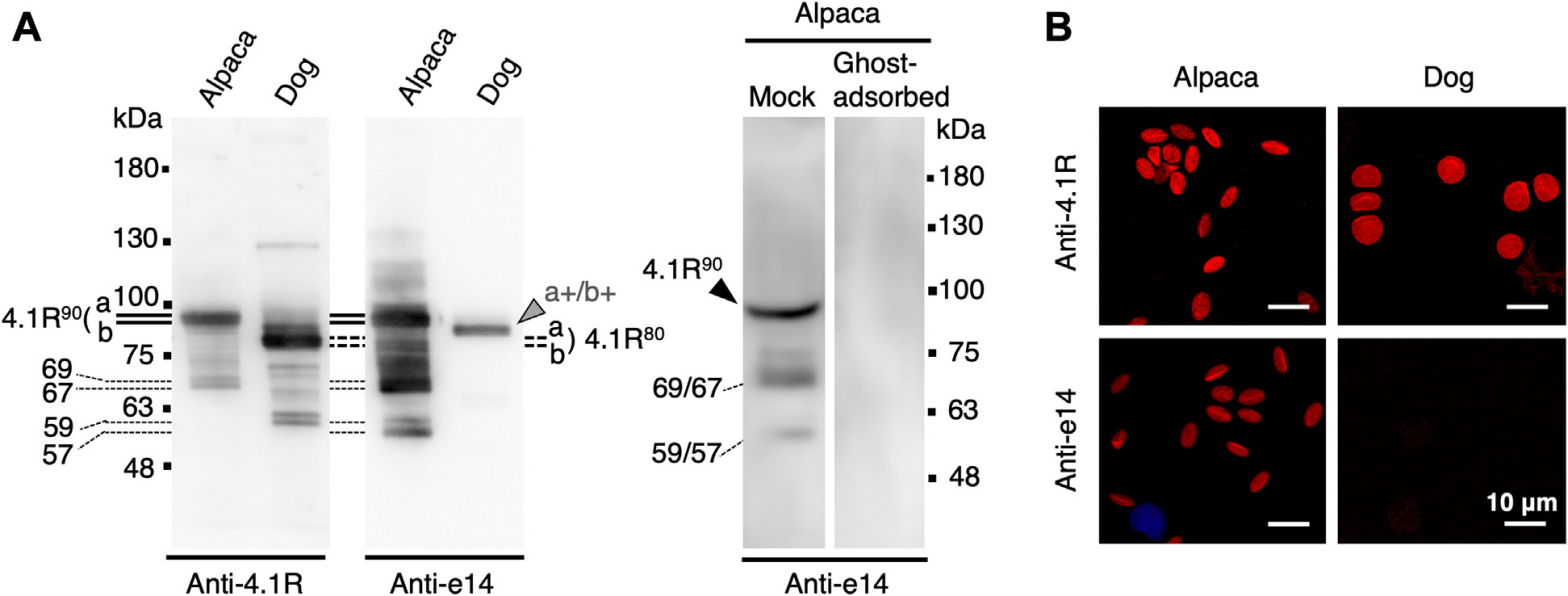
**Immunoblotting and immunofluorescent detection of the exon 14–derived sequence in alpaca 4.1R**^**90**^**.***A*, immunoblotting analysis of 4.1R proteins from alpaca and dog RBC membranes using anti-canine 4.1R (*Anti-4.1R*) and anti-e14 (*Anti-e14*) antibodies. Alpaca 4.1R^90^ reacted with both antibodies, whereas dog 4.1R^80^ reacted only with anti-4.1R antibody. The anti-e14 antibody reacted weakly with proteins slightly larger than 4.1R^80^ (*a+/b+*) in dog membranes, indicating the presence of a 4.1R^80^ isoform having the e14 sequence at a very low level. Each lane contained 3 μg (*alpaca*) and 2 μg (*dog*) membrane proteins (*left panels*). The *right panel* shows the immunoblotting of alpaca RBC membranes with anti-e14 antibody previously incubated with (*Ghost-adsorbed*) or without (*Mock*) an excess amount of alpaca RBC membranes, as described in the Experimental procedures. The migrating positions of size markers are shown in kDa. *B*, immunofluorescent detection of 4.1R protein in alpaca and dog RBCs showing the involvement of the e14 sequence in alpaca RBC membranes. Bars represent 10 μm. RBC, red blood cell.

MS/MS analysis of SDS-PAGE-purified 4.1R^90^ and a database search detected the amino acid sequences from all the exons comprising 4.1R^90^ cDNA, except that no peptides encoded by exon 19 were detected (Fig. S2), presumably due to the *O*-linked GlcNAc modification of some of the threonine or serine residue(s) in the exon 19–derived region (36). The tryptic peptides with the sequences ^388^KEE…EK^417^ or ^389^EE…EK^417^ derived from the PE, and the sequence ^421^TH…QK^437^ derived from e14, were detected in several independent analyses, demonstrating the involvement of these sequences in alpaca 4.1R^90^. Immunoblotting of RBC membranes and immunofluorescent staining of RBCs with antibody raised against the e14 cassette further confirmed the unusual expression of the exon 14–derived sequence in alpaca 4.1R^90^ (Fig. 3, *A* and *B*). The anti-e14 antibody detected several minor bands other than 4.1R^90^ in alpaca RBC membranes; some, including the 69/67 kDa doublet, were also recognized by anti-4.1R, whereas others, such as the 59/57 kDa polypeptides, were not. These polypeptides are most likely minor isoforms of 4.1R that contain the e14 cassette but lack other regions, as these bands, as well as the 4.1R^90^ doublet, totally disappeared when the antibody was incubated with excess amounts of leaky ghosts from alpaca RBCs and subsequently used for detection (Fig. 3*A*, right panel). In contrast, a very low level of e14 cassette expression was detected in a minor isoform of 4.1R in canine RBC membranes (*4.1Ra+/b+* in Fig. 3*A*), a finding consistent with the very weak expression of exon 14 in late-stage human erythroblasts and reticulocytes (15, 16, 35).

### The PE–e14 sequence in alpaca 4.1R^90^ strengthens membrane skeletal complex formation

**Figure 4:**
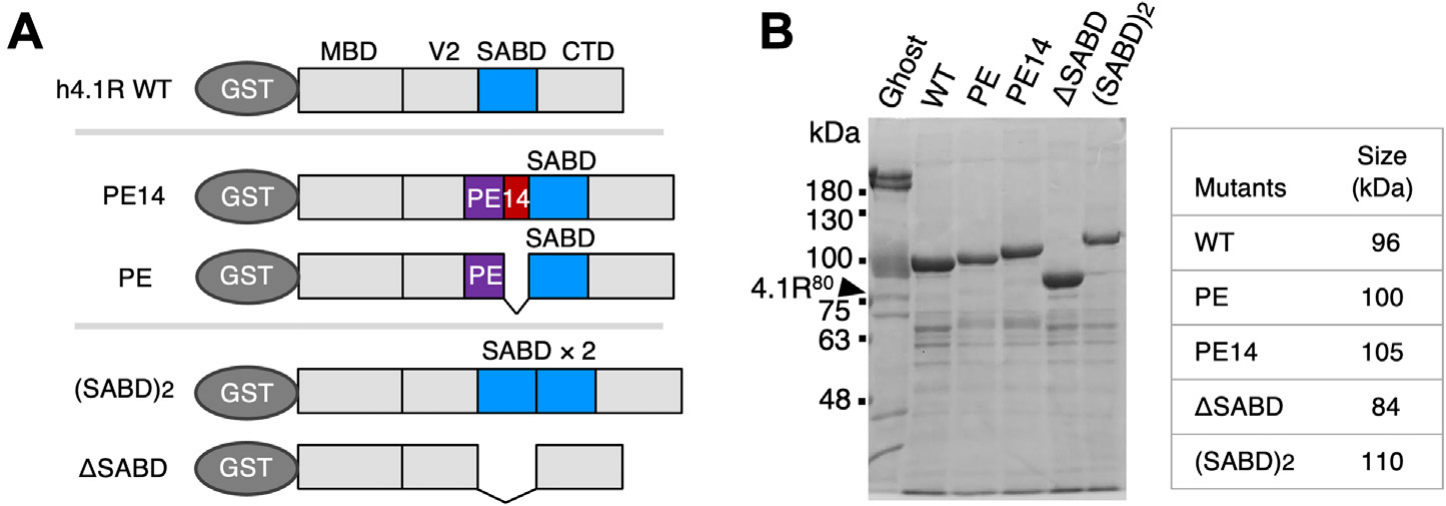
**GST-fused h4.1R mutants used in the gelation assay.***A*, schemes of h4.1R WT and its mutants used in gelation assays. Mutants included *PE14*, h4.1R[PE14]; *PE*, h4.1R[PE]; *(SABD)*_*2*_, h4.1R(SABD)_2_; and *ΔSABD*, h4.1RΔSABD. Assignment of structural domains is described in the legend for Figure 2. *B*, Coomassie blue–stained SDS-gel of the purified recombinants. Human RBC membrane proteins are also shown (*Ghost*), with 4.1R^80^ (*4.1R*^*80*^) indicated. The apparent molecular masses of GST-fused recombinants and size markers are shown in kDa (*right panel*). GST, glutathione-S-transferase; RBC, red blood cell; SABD, spectrin–actin-binding domain.

To determine if the PE and e14 sequences affect membrane skeletal formation, the effects of these sequences on spectrin–actin–4.1R ternary complex formation were analyzed using the falling ball viscometry assay, employing the glutathione-S-transferase (GST)-fused WT of human 4.1R (h4.1R) and h4.1R mutants containing the PE (h4.1R[PE]) or both the PE and e14 (h4.1R[PE14]) sequences (Fig. 4). This analysis also included a mutant h4.1R(SABD)2, which contained the SABD in tandem duplication and mimicked a mild HE-causing mutant 4.1^95^ (30, 31), as well as the h4.1RΔSABD mutant that lacked the SABD. Inclusion of the PE and e14 sequences added a total of 31 amino acid residues, theoretically increasing the molecular mass of 4.1R by approximately 3.5 kDa. However, the molecular masses of GST-fused h4.1R[PE14] and h4.1R(SABD)2 on SDS-PAGE were approximately 10 kDa or 15 kDa larger, respectively, than GST-WT 4.1R^80^, consistent with the differences between the molecular mass of 4.1R^80^ and the molecular masses of 4.1R^90^ or 4.1R^95^, respectively (Fig. 4*B*). The increased negative charge and unordered structure of the PE14 sequence might contribute to the reduced mobility of the protein on SDS-PAGE and the discrepancy between the 3.5 kDa difference in theoretical mass change and the 10 kDa difference in apparent size.

**Figure 5:**
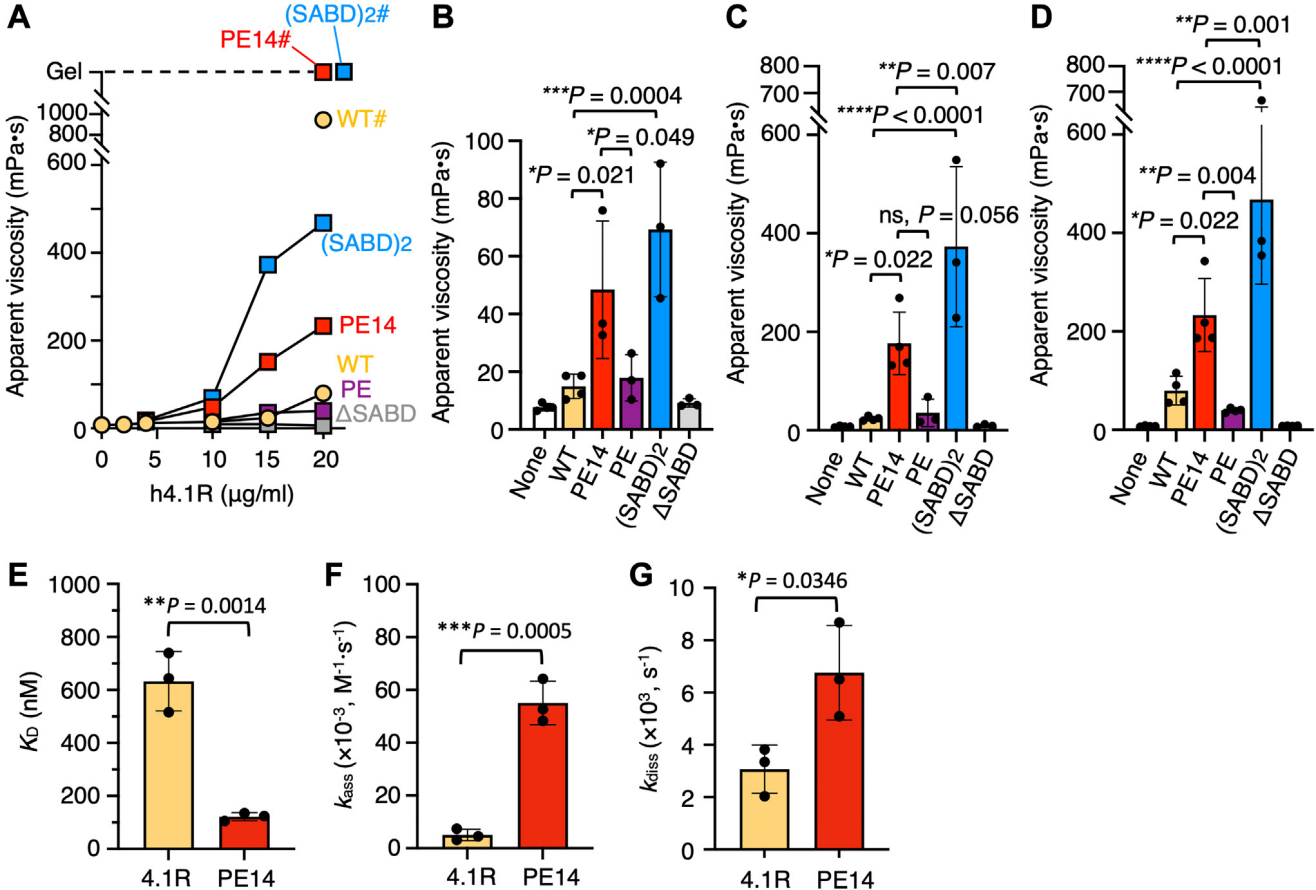
**Effects of the PE14 sequence on spectrin–actin–4.1R ternary complex formation and 4.1R binding to the N-terminal region of β-spectrin.***A*, results of falling ball viscometry assays, showing the apparent viscosity due to the spectrin–actin–4.1R complex formation. Each reaction contained 10 μg/ml spectrin tetramers, 250 μg/ml F-actin, and appropriate concentrations (0–20 μg/ml) of GST-fused h4.1R WT or the h4.1R mutants shown in Figure 3. Data shown are the means of three independent experiments. The viscosities obtained for the WT (*WT#*) and the PE14 (*PE14#*) and (SABD)_2_ (*(SABD)*_*2*_*#*) recombinants in the presence of 20 μg/ml spectrin tetramers are also shown. PE14# and (SABD)_2_# reactions showed complete gelation of the solutions (*Gel*). *B–D*, detailed data and comparisons for reactions containing 10 (*B*), 15 (*C*), and 20 μg/ml (*D*) h4.1R recombinants. *None* indicates that the reaction mixture contained only spectrin and F-actin. Data are expressed as the mean ± S.D. (n = 3–4). *p*-values are calculated by one-way ANOVA with Dunnett’s multiple comparison test. *E–G*, results of surface plasmon resonance assays, showing the binding affinities of h4.1R WT (*WT*) and h4.1R[PE14] (*PE14*) to the N-terminal fragment of β-spectrin. *K*_D_ values (*E*) are calculated from the measurements of *k*_ass_ (*F*) and *k*_diss_ (*G*). Data are expressed as the mean ± S.D. (n = 3). *p*-values calculated by unpaired *t* tests. GST, glutathione-S-transferase; SABD, spectrin–actin-binding domain.

The addition of the WT h4.1R, but not the ΔSABD mutant h4.1R, at increasing concentrations up to 20 μg/ml increased the viscosity of the solution of 10 μg/ml spectrin tetramers and 250 μg/ml F-actin, confirming that the increase in viscosity was induced by spectrin–F-actin–4.1R complex formation through binding of 4.1R to spectrin and actin at the SABD (Fig. 5, *A*–*D*). Under the same conditions, h4.1R[PE14] markedly increased viscosity to a level significantly higher than that induced by WT h4.1R. In contrast, the viscosity of solution containing the h4.1R[PE] mutant did not differ significantly from the viscosity of solution containing WT h4.1R. These findings indicate that the e14 sequence substantially increases the stability of spectrin–actin–4.1R binding at the SABD. In addition, h4.1R(SABD)2 markedly increased ternary complex formation, resulting in about a two-fold higher viscosity than that of the complex formed by h4.1R[PE14]. Furthermore, in the presence of 20 μg/ml spectrin, the addition of the WT h4.1R markedly increased, whereas both h4.1R[PE14] and h4.1R(SABD)_2_ caused complete gelation of the solution (Fig. 5*A*).

Compared with WT h4.1R, h4.1R[PE14] had a five-fold lower equilibrium dissociation constant (*K*_D_) for binding to the N-terminal polypeptide of β-spectrin (121 ± 15 nM vs. 633 ± 112 nM, *p* < 0.01, n = 3 each; Fig. 5*E*). The association rate constant (*k*_ass_) of h4.1R[PE14] was significantly higher than that of WT h4.1R ((55.07 ± 8.21) × 10^3^ M^−1^ s^−1^ vs. (5.04 ± 2.16) × 10^3^ M^−1^ s^−1^, *p* < 0.001, n = 3 each; Fig. 5, *F* and *G*). These findings account for the higher β-spectrin binding affinity of h4.1R[PE14] than of WT h4.1R.

## Discussion

The present study showed that camelid 4.1R is unique among RBC 4.1R proteins in that it contains the e14 cassette that is absent from 4.1R^80^, the major 4.1R isoform in RBC membranes of other mammalian species. Functionally, the inclusion of e14 markedly increased the formation of the ternary complex of 4.1R, spectrin, and actin through association at the SABD. The gelation activity of SABD in this assay correlated with its ability to strengthen membrane integrity (21). Moreover, erythroid-specific expression of the 21-aa cassette from exon 16 has been implicated in the highly efficient interactions of the SABD with spectrin and F-actin necessary to maintain the mechanical stability of RBC membranes, with a reduced yet adequate binding activity being due to the constitutive 59-aa exon 17–coded region alone (17, 21). Thus, e14 appears to provide camelid 4.1R^90^ the ability to form hyperstable spectrin–actin–4.1R ternary complexes at the junctions in the RBC membrane. Quantification of alternative exon expression indicated that the percentage of the +14/+16 isoform, 4.1R^90^, increases as cells mature from erythroblasts to reticulocytes, consistent with a model indicating that this isoform alters membrane properties in mature RBCs.

The membrane skeleton undergoes rearrangement through repeated dissociation and reassociation of spectrin tetramers and dimers, respectively, enabling the RBC membrane to accommodate shear stress-induced distortion in the circulation (27, 37, 38). Prolonged membrane skeleton rearrangement with a subsequent deformed configuration precludes the recovery to the biconcave shape and results in the formation of irreversibly deformed RBCs, such as sickled cells and elliptocytes in HE (4, 5, 26, 27). In addition, the N-terminal fragment of β-spectrin, containing the binding sites for 4.1R and actin, was shown to be incorporated into junctions in intact membranes, indicating that the ternary complex in the junctions is somewhat labile (39, 40). The hyperstability of the spectrin–actin–4.1R^90^ interaction suggests the rearrangement of RBC membrane skeletons with reduced plasticity, through the formation of stable junctions, and facilitated the reassociation of spectrin dimers in a deformed configuration, leading to the formation and maintenance of rigid elliptocytes. This scenario can explain the change in shape from sphere to ellipsoid during reticulocyte maturation observed in the present study and the absence of deformative responses of camelid elliptocytes to shear stress (7). The present study also showed that the human 4.1R^95^ variant resulting from the SABD duplication mutation was associated with the pathogenesis of HE (30, 31). The markedly increased viscosity of the ternary complex resulting from the addition of h4.1R(SABD)_2_ strongly suggests that the hyperstable junctions containing the 4.1R^95^ variant bestow increased membrane integrity in this HE subtype (30, 32), presumably by increasing the number of binding sites for spectrin and actin at the junctions. Thus, camelid 4.1R^90^ and the human 4.1R^95^ variant exemplify membrane-associated proteins whose structural changes result in rigid and hyperstable RBCs, similar to a band 3 mutant in Southeast Asian ovalocytosis (41, 42).

The results of this study also suggest that the e14 cassette increases the binding affinity of 4.1R to β-spectrin, as demonstrated by a surface plasmon resonance measurement assay for the binding of h4.1R mutants to the N-terminal fragment of β-spectrin, suggesting a possible mechanism underlying the increased stability of ternary complex formation. Secondary structure prediction using several algorithms, such as JPred4 (http://www.compbio.dundee.ac.uk/jpred4) and CRNPRED (http://pdbj.org.crnpred), showed that the amino acids derived from exons 13–14, including the PE14 sequence, do not form an appreciably ordered structure. However, e14 may fold upon binding of SABD to its partner proteins spectrin and actin, stabilizing their association. Similar findings have been reported for the functions of several intrinsically disordered proteins, including the N-terminal headpiece of the erythroid isoform 4.1R^135^ (43, 44). These findings, however, are inconsistent with previous results analyzing the interaction of several SABD constructs (20). These analyses showed that the N-terminal addition of the e14 sequence to the SABD reduced its affinity to spectrin and F-actin due to steric hindrance (20). These discrepancies may be due to differences in the SABD constructs used in these studies, especially the use of whole h4.1R proteins in the present study. Alternatively, the disordered PE sequence confers some role on the effect of e14, although PE itself is unable to form hyperstable ternary complexes.

Hyperstable junctions may also affect the interaction of the membrane skeleton with the lipid bilayer. Band 3 and glycophorin C were each shown to account for 50% of total membrane-bound 4.1R, with the dissociation of 4.1R from band 3 increasing band 3–ankyrin interactions, strengthening the bilayer–skeleton association (45). Notably, *K*_D_ values for the binding of 4.1R to band 3 and glycophorin C were very similar in the submicromolar (10^−7^ M) range (46). Therefore, the increased affinity of β-spectrin to 4.1R containing the PE14 sequence may increase the proportion of 4.1R bound to spectrin and actin at the junctions and, by increasing the dissociation of 4.1R from band 3, increase the band 3–ankyrin interaction, which has an affinity of 10^−7^–10^−8^ M (45), slightly higher than 4.1R–band 3/glycophorin C binding. This hypothesis is consistent with the results of the present study and an earlier study (24) showing that most of the band 3 in camelid RBC membranes is retained in the precipitate upon solubilization with Triton X-100, presumably because most of the band 3 is bound to the membrane skeleton in circulating RBCs. The change in binding partners of band 3 from 4.1R to ankyrin has also been shown to decrease membrane deformability and increase membrane mechanical stability (45), compatible with the properties of camelid RBC membranes.

Decreased membrane deformability could be disadvantageous for animals. However, the size (mean corpuscular volume) of camelid RBCs ranges from 21 to 28 fl (47) and is smaller than the RBCs of most other mammals, including humans. The ellipsoid shape and small size may allow camelid RBCs to pass through the microvasculature without significant deformation. However, camelid RBCs are also characterized by their thin and flattened shape and have an average hemoglobin concentration (mean corpuscular hemoglobin concentration, MCHC) higher than that of RBCs in other mammals (47). This is consistent with findings showing that high concentrations of hemoglobin restrict the diffusion of CO2 and HCO3^−^ inside RBCs, which is a strong rate-limiting factor for gas turnover (48). Moreover, RBC thickness was found to be reduced as appropriate for their MCHC to compensate for highly restricted cytoplasmic diffusion (48). Earlier studies suggested that morphogenesis of avian RBCs during reticulocyte maturation occurs in two distinct steps, marginal band microtubule-dependent flattening and subsequent ellipsoid formation, by as yet undefined mechanisms (10, 11). The marginal band is also seen in camelid reticulocytes, but not in erythrocytes (9), suggesting that a similar flattening of marginal bands precedes ellipsoid transformation. Therefore, 4.1R^90^ likely participates not only in the generation and maintenance of an ellipsoid shape but also in the maintenance of a flattened shape of camelid RBCs, possibly through some intricate three-dimensional interaction of skeletal proteins in equatorial locations. Camelid RBCs are also characterized by their resistance to hypotonic lysis (7, 49, 50) and to dehydration in hypertonic solution without significant shape changes (24, 51). These properties allow camelids to withstand dehydration for extended periods of time and to tolerate rapid ingestion of large volumes of water. The increased band 3 content and protein:lipid ratio on camelid RBC membranes may also be involved in this characteristic osmotic resistance (24, 49). Hyperstabilization of the membrane skeleton may also contribute to increased osmotic resistance by strengthening the membrane–membrane skeleton association, as described above.

These findings indicate that the expression of the alternative exon 14 is likely an evolutionary adaptation of camelids to maintain facilitated gas turnover in concert with some genetic traits of RBCs, including mean corpuscular volume and MCHC (52). The expression of the alternative exon 14 has been found in early erythroblasts in humans, resulting in one of the major isoforms of 4.1R^135^, although expression of this exon is decreased and restricted to a very minor population of 4.1R^80^ in late erythroblasts and reticulocytes (15, 16). Exon 14 expression is also seen in the brain and endothelial cells (15, 53). The functional significance of the exon 14–derived sequence in some 4.1R isoforms is unknown. Because the amino acid sequences encoded by exons 14, 16, and 17 are highly conserved among various mammalian species (Fig. S3), the exon 14 element likely plays some modulatory role in the tissue- and development-specific regulation of skeletal proteins.

In conclusion, the present study described a characteristic feature of camelid 4.1R^90^, suggesting the involvement of this protein in elliptocyte formation in camelid species. These findings also indicate that alternative splicing of exons may play a major role in regulating structural organization and membrane function due to the multifunctional skeletal protein 4.1R.

## Experimental procedures

### Animals

Adult male alpacas and dogs were housed at the animal experimentation facility of the Graduate School of Veterinary Medicine, Hokkaido University. All experimental procedures were reviewed and approved by the Laboratory Animal Experimentation Committee, Graduate School of Veterinary Medicine, Hokkaido University with an approval number 16-0085.

### Materials

Antibodies to canine 4.1R, spectrin, and band 3 have been described previously (18, 34, 54). Rabbit antibody to the exon 14–derived sequence (anti-e14) was generated by the injection of a synthetic peptide with the amino acid sequence of NH2-^419^VEKTHIEVTVPTSDGDQTQ^437^-COOH. The amino acid sequence encoded by alternative exon 14 is identical in various species, including alpacas, dogs, and humans (Fig. S3). The Asn^432^ residue deduced from the cDNA sequence was converted to Asp (underlined) because Asp was present in the peptides detected by MS/MS analysis (Table S1), presumably due to a rapid posttranslational deamidation at the Asn-Gly sequence (19). In some experiments, the anti-e14 antibody was incubated with an excess amount of alpaca RBC membrane ghosts (200 μg membrane protein/μg antibody) in 10 mM Tris–Cl (pH 7.5), 500 mM NaCl on ice for 2 h followed by sedimentation of the ghosts by centrifugation. The antibody was subjected to mock treatment by adding it to the ghosts just before centrifugation.

Spectrin tetramers were prepared as described (55, 56). Briefly, spectrin was extracted from dog RBC membranes and subjected to gel permeation chromatography on a Sepharose 4B column to separate dimers and tetramers. Fractions containing dimers were collected, concentrated by ultrafiltration, and incubated at 30 °C for 2 h to promote conversion of dimers to tetramers. The samples were again subjected to gel permeation chromatography, and the tetramers were pooled and concentrated. Rabbit muscle actin was purchased from Cytoskeleton Inc.

### Analysis of alpaca RBC shapes

The larger and smaller diameters of RBCs were measured on new methylene blue–stained smears of alpaca peripheral blood. RBCs were grouped into younger aggregate reticulocytes, maturing punctate reticulocytes, and erythrocytes. Ellipticity was calculated as (a−b)/a values, where a and b are the larger and smaller diameters, respectively, of RBCs.

The shape of RBC membrane skeletons (Triton shell) was examined under light microscopy after solubilization of the membrane in PBS containing 1% Triton X-100 for 30 min on ice.

### LC-MS/MS analysis

The amino acid sequence of alpaca RBC 4.1R was determined by Fourier transform-MS/MS, as reported previously (57). Briefly, alpaca RBC membrane proteins were separated on 8% SDS-PAGE gels and stained with Coomassie brilliant blue. The gel slices containing 4.1R polypeptides (∼90 kDa) were excised and subjected to reduction with 10 mM DTT, alkylation with 55 mM iodoacetamide, and digestion with 10 μg/ml of trypsin Gold (Promega) for 16 h at 37 °C. The tryptic peptides were extracted in 50% acetonitrile/5% TFA and concentrated by evaporation. The peptides dissolved in 0.1% TFA were separated and analyzed on a tandemly connected Dionex UltiMate 3000 liquid chromatography system and LTQ Orbitrap Fourier transform-MS/MS system (Thermo Fisher Scientific). *De novo* sequencing, comparison with the alpaca reference genome in the NCBI VicPac3.1 database, and posttranslational modification analysis were performed using PEAKS 7 software (https://www.bioinfor.com/peaks-online/, Bioinformatics Solutions Inc).

### Alpaca erythrocyte 4.1R cDNA

Heparinized blood obtained from alpacas was filtered through an α-cellulose/microcrystalline cellulose column to remove leukocytes and platelets (18). mRNA was purified from the resultant RBCs containing reticulocytes, and from erythroblasts amplified from precursor cells in peripheral blood, and was reverse-transcribed into cDNA. Peripheral blood mononuclear cells were cultured in two-phase liquid culture as described (58). After 6 days in the second phase, the cells (day 6 erythroblasts, consisting mainly of basophilic and polychromatophilic erythroblasts) were harvested, and mRNA was prepared. The 4.1R cDNA fragments were amplified by PCR using the primer pairs Vic41.e4.F and Vic41.e21.R, Vic41.e11.PSYRAA.F and Vic41.e17.KKHHASI.R, or Vic41.e12.TRQASA.F and Vic41.e17.VPEPRP.R (Table S2), cloned, and sequenced. Two independent allelic sequences of cDNAs obtained were deposited in GenBank with accession numbers OM890907 and OM890908. The expression of alternative exons in reticulocytes and day 6 erythroblasts was analyzed by PCR using the primers Vic41.5′e13PE.F, Vic41.e17.KKHHASI.R, Vic41.e12.TRQASA.F, and Vic41.seq.e16.R2 (Table S2), followed by direct sequencing and digestion of the amplified cDNA fragments with *Xsp* I or *Taq* I. The relative abundance of cDNA fragments separated by electrophoresis was determined using ImageJ software (imagej.nih.gov/ij/download/, National Institute of Health).

### Plasmids

The plasmids pGST-h4.1R and pGST-βspeN were kindly provided by Shotaro Tanaka, Ichiro Koshino, and Yuichi Takakuwa of Tokyo Women’s Medical University. pGST-h4.1R contained the WT h4.1R cDNA fused to the N-terminal GST in the pGEX-6P-3 (Cytiva) backbone. pGST-βspeN contained the GST-fused N-terminal fragment (amino acid residues 1–527) of human β-spectrin in the pGEX-4T-2 (Cytiva).

The plasmid for the expression of GST-fused h4.1R lacking SABD (pGST-h4.1ΔSABD) was prepared by inverse PCR of pGST-h4.1R using the primers h41R3′e13.R and h41R5′e18.F (Table S2), followed by ligation. Mutant h4.1R(SABD)2 with duplicated SABD, mimicking the mild HE-causing mutant 4.1^95^ (30, 31), was also prepared. The cDNA fragment of exons 16–18 encoding ^407^K–Q^529^ was amplified from pGST-h4.1R by PCR using the primers h41R5′e16.F and h41R3′e18.R. This fragment was ligated into the vector fragment obtained by inverse PCR of pGST-h4.1R using the primers h41R3′e13.R and h41R5′e16.F; the plasmid pGST-h4.1R(SABD)2 containing duplicated ^407^K–Q^529^ sequences was also prepared.

To generate h4.1R recombinants containing an alpaca 4.1R-specific PE/e14 sequence, the cDNA fragment was amplified by PCR using the primers Vic41.5′e13PE.F and Vic41.3′e14.R and ligated into the vector fragment obtained by inverse PCR of pGST-h4.1R with the primers h41prePE.R and h41R5′e16.F. The resultant plasmid pGST-h4.1R[PE14] was used for the expression of the h4.1R recombinant, in which the C-terminal region of the V2 domain (^391^D–K^406^) was replaced by the PE and e14 sequences derived from alpaca 4.1R. The pGST-h4.1R[PE14] was further amplified by inverse PCR with the primers Vic41.3′e13PE.R and Vic41.5′e16.F3, and the proximal ends were ligated to prepare pGST-h4.1R[PE], from which the e14 sequence had been removed.

### Preparation of recombinant proteins

*Escherichia coli* BL21(DE3)pLysS competent cells (Promega) were transformed with the appropriate pGEX vectors, and protein expression was induced by culturing the cells with 1 mM IPTG for 6 h at 30 °C. Bacterial cells were collected and lysed in the B-PER reagent (Thermo Fischer Scientific). GST-fused h4.1R recombinant proteins in the lysate were trapped on the glutathione Sepharose 4B resin (Cytiva) and eluted with 10 mM reduced GSH.

To measure surface plasmon resonance, some h4.1R recombinants captured on the glutathione Sepharose beads were cleaved from GST by the addition of PreScission protease (Cytiva) and eluted from the beads. The proteins were further purified by anion exchange chromatography on a MonoQ column (Cytiva) based on the procedure used to purify RBC 4.1R (19, 36).

The GST-fused N-terminal fragment of human β-spectrin (GST-hNβSp) was generated in BL21(DE3)pLysS cells transformed with pGST-βspeN and purified on a glutathione Sepharose column as described above.

### Falling ball viscometry assay

Spectrin–actin–4.1R ternary complex formation was measured by the falling ball viscometry assay, as described (59, 60). In brief, spectrin, F-actin, and GST-h4.1R mutants at appropriate concentrations were mixed in 10 mM Tris–Cl (pH 7.6), 20 mM KCl, 2 mM MgCl2, and 1 mM ATP. A 100 μl microcapillary tube (Drummond Scientific) was filled with this solution, sealed at one end, and incubated at the indicated temperature. After incubation, the tube was placed at a 70° angle from the horizontal; a steel ball (diameter 0.6 mm, Tsubaki-Nakashima Co) was placed under the meniscus of the solution using a magnet; and the time required by the ball to fall a specific distance (6 cm) at 25 °C was measured. Sedimentation velocity was calculated from the falling time (F_t_, s/cm) and converted to apparent viscosity (mPa·s) using glycerol standards of known viscosity. The apparent viscosity of reactions containing only spectrin and F-actin was used as the control. If the ball did not move from its initial position for 120 s, the protein mixture was thought to form a gel. In most measurements, spectrin and F-actin concentrations were 10 and 250 μg/ml, respectively; to this, GST-h4.1R recombinants were added at the concentrations of 0 to 20 μg/ml, yielding a spectrin:actin:4.1R M ratio of 1:125:0–5.

### Binding assay by surface plasmon resonance measurement

Interactions between recombinant h4.1R (h4.1R WT and h4.1R[PE14]) and GST-hNβSp were analyzed by measuring surface plasmon resonance on the Biacore X100 system (Cytiva). GST-hNβSp or GST alone as the control, at concentrations of 500 nM, was immobilized on the surface of CM5 sensor chips (Cytiva) using an anti-GST antibody (MBL). Association and dissociation analyses of recombinant proteins at appropriate concentrations (150–1000 nM) were performed in PBS at 25 °C. At the end of each measurement, the chip was regenerated in 10 mM Glycine–Cl (pH 2.1). Data were processed with the Biacore X100 evaluation software (https://www.cytivalifesciences.com/en/us/support/software/biacore-downloads/biacore-x100-software, Cytiva).

### Analyses of proteins

RBC membranes were prepared, and SDS-PAGE and immunoblotting were performed, as previously described (18, 36). Protein concentrations were measured and immunofluorescence microscopy was performed as described previously (57).

### Statistical analysis

All data are expressed as the mean ± S. D. Statistical significance was determined by unpaired Student *t* tests or one-way ANOVA with Tukey’s or Dunnett’s multiple comparison test. All statistical analyses were performed by using GraphPad Prism 9.0 (GraphPad), with a *p*-value < 0.05 considered statistically significant.

## Data availability

Alpaca DNA sequences were deposited in GenBank under accession numbers OM890907 and OM890908. All other data are contained within the article and supporting information.

## Supporting information

This article contains supporting information (19, 35, 54).

## Conflict of interest

The authors declare that they have no conflicts of interest with the contents of this article.
