# Supplementary material for: Membrane skeleton hyperstability due to a novel alternatively spliced 4.1R can account for ellipsoidal camelid red cells with decreased deformability
Source: J Biol Chem. 2023 Jan 6;299(2):102877. doi: 10.1016/j.jbc.2023.102877 (PMC9926112; doi:10.1016/j.jbc.2023.102877)
Supplement: Supplemental Tables S1–S2 and Figures S1–S3 [file mmc1.docx]

**Supporting Information**

**Membrane skeleton hyperstability due to a novel alternatively spliced 4.1R can account for ellipsoidal camelid red cells with decreased deformability**

Yuqi Chen^1^, Kosuke Miyazono^1^, Yayoi Otsuka^1^, Mariko Kanamori^1^, Aozora Yamashita^1^,

Nobuto Arashiki^1,2^, Takehisa Matsumoto^3^, Kensuke Takada^1^, Kota Sato^1^, Narla Mohandas^4^, and Mutsumi Inaba^1,^*

^1^Laboratory of Molecular Medicine, Graduate School of Veterinary Medicine, Hokkaido University, Sapporo 060-0818, Japan

^2^Department of Biochemistry, School of Medicine, Tokyo Women’s Medical University, Tokyo 162-8666, Japan

^3^Laboratory of RIKEN Center for Biosystems Dynamics Research, Yokohama 230-0045, Japan.

^4^Red Cell Physiology Laboratory, New York Blood Center, New York, NY 10065, U.S.A.

*Corresponding author

**Table S1. An example of LC-MS/MS data for alpaca 4.1R^90^**

An example of database searching for the LC-MS/MS analysis of alpaca 4.1R^90^ separated on SDS-gels. The peptides corresponding to the PE and e14 sequences are highlighted.

| Peptide | −10lgP | PTM* |
| --- | --- | --- |
| R.SPRPTSAPAIAQSEVTEGSVPGAPVKK.A | 70.27 |  |
| K.^421^THIEVTVPTSN(+.98)GDQTQK^437^.K | 67.49 | Deamidation (NQ) |
| R.SM(+15.99)TPAQADLEFLENAK.K | 65.25 | Oxidation (M) |
| R.HSNLMLEDLDK.S | 63.18 |  |
| K.IRPGEQEQYESTIGFK.L | 62.94 |  |
| K.VSLLDDTVYEC(+57.02)VVEK.H | 60.9 | Carbamidomethylation |
| R.SMTPAQADLEFLENAK.K | 59.88 |  |
| R.SLDGAAVDSADR.S | 58.17 |  |
| K.TQTVTITDTANAVK.S | 58.17 |  |
| R.LDGENIYIR.H | 57.29 |  |
| R.HSNLM(+15.99)LEDLDK.S | 54.54 | Oxidation (M) |
| K.^388^KEEVPAEPAEPAEPPEPPEPPEPEPTEVEK^417^.V | 51.46 |  |
| K.NFM(+15.99)ESVPEPR.P | 51.25 | Oxidation (M) |
| R.LSTHSPFR.T | 51.08 |  |
| K.DVPIVHTETK.T | 50.54 |  |
| K.NFMESVPEPR.P | 49.97 |  |
| K.VVVHQETEISED | 48.06 |  |
| R.IVITGDADIDHDQVLVQAIK.E | 47.65 |  |
| R.QASALIDRPAPH.F | 47.02 |  |
| R.S(+79.97)LDGAAVDSADR.S | 46.97 | Phosphorylation (STY) |
| K.TQTVTITDTAN(+.98)AVK.S | 43.64 | Deamidation (NQ) |
| K.HHASISELK.K | 42.64 |  |
| K.QVHGIPWNFTFNVK.F | 42.52 |  |
| K.^421^THIEVTVPTSNGDQ(+.98)TQK^437^.K | 41.58 | Deamidation (NQ) |
| K.KHHASISELKK.N | 41.35 |  |
| K.TQ(+.98)TVTITDTANAVK.S | 39.65 | Deamidation (NQ) |
| K.HHASISELKK.N | 39.64 |  |
| R.LTSTDTIPK.S | 38.83 |  |
| K.FLALGSK.F | 37.59 |  |
| R.QASALIDRPAPHFER.T | 36.92 |  |
| K.TWLDPAK.E | 36.01 |  |
| K.RLSTHSPFR.T | 35.29 |  |
| K.^389^EEVPAEPAEPAEPPEPPEPPEPEPTEVEK^417^.V | 34.93 |  |
| R.INRFPWPK.V | 29.63 |  |
| R.PTSAPAIAQSEVTEGSVPGAPVKK.A | 28.79 |  |
| R.SMTPAQAD(+15.99)LEFLENAK.K | 28.19 | Hydroxylation |
| R.SPRPTSAPAIAQSEVTEGSVPGAPVK.K | 24.31 |  |
| K.EQHPDMSVTK.V | 23.55 |  |

*PTM, possible posttranslational modification

**Table S2. Primers used in the present study**

| Primer | Nucleotide sequence | Purpose |
| --- | --- | --- |
| Vic41.e4.F  Vic41.e21.R  Vic41.e11.PSYRAA.F  Vic41.e17.KKHHASI.R  Vic41.e12.TRQASA.F  Vic41.e17.VPEPRP.R  Vic41.e13.AIAQSEV.F  Vic41.e17.RPSEWD.R  h41R.3'e13.R  h41.5'e18.F  h41.5'e16.F  h41.3'e18.R  Vic41.5'e13PE.F  Vic41.3'e14.R  h41.prePE.R  Vic41.3'e13PE.R  Vic41.5'e16.F3  Vic41seq.e8.F  Vic41seq.e8.R  Vic41seq.e10.R  Vic41seq.e14.F  Vic41seq.e14.F2  Vic41seq.e16.R  Vic41seq.e16.R2  Vic41seq.e20.R  Vic41seq.e21.R | 5'-GCTTCCCAGAAATCAATCAGA-3'  5'-CAGTCCTCAGAGATTTCGGTCT-3'  5'-CCCAGTTACCGAGCAGCTAA-3'  5'-TGATGCTGGCATGATGTTTT-3'  5'-GACCAGGCAAGCTAGTGCTC-3'  5'-TAGGCCGTGGTTCTGGTACA-3'  5’- GCCATTGCTCAGCGTGAGGT-3’  5’-TTATCCCATTCGCTAGGCCG-3’  5'-CTTCCATGCTTCTGTGGGCTCTG-3'  5'-CCTCCCCTGGTGAAGACACA-3'  5'-AAAAAGAGAGAAAGACTAGA-3'  5'-CTGGGCAGCCTCATAAGTGA-3'  5'-GAAGTGCCAGCTGAGCCAG-3'  5'-CTGTGTTTGGTCACCATTTGA-3'  5'-TTCCTTTTTCACTTCAGCCTTCACTGT-3'  5'-CTTCTCCACTTCTGTGGGCTC-3'  5'-AAAAAGAGAGAAAGACTAGATGGTGAAAAC-3'  5'-GTCTTCAACTTCGGCAGGAC-3'  5'-TGGGTCATAGTCTCCCAGCT-3'  5'-GGCCGAATCTTGATGAAAAA-3'  5'-AAAACCCACATCGAGGTCAC-3'  5'-CCTCAAATGGTGACCAAACA-3'  5'-AAATCCTCCAACATTAAATTGCT-3'  5'-TGTTTTCACCATCTAGTCTCTCTCTC-3'  5'-GGGTCTCTGAAATTCCACCTT-3'  5'-CAGTCCTCAGAGATTTCGGTCT-3' | cDNA cloning  cDNA cloning  cDNA cloning/qPCR  cDNA cloning/qPCR  cDNA cloning/qPCR  cDNA cloning  qPCR  qPCR  Plasmid construction  Plasmid construction  Plasmid construction  Plasmid construction  Plasmid construction/qPCR  Plasmid construction  Plasmid construction  Plasmid construction  Plasmid construction  DNA sequencing  DNA sequencing  DNA sequencing  DNA sequencing  DNA sequencing  DNA sequencing/qPCR  DNA sequencing/qPCR  DNA sequencing  DNA sequencing |


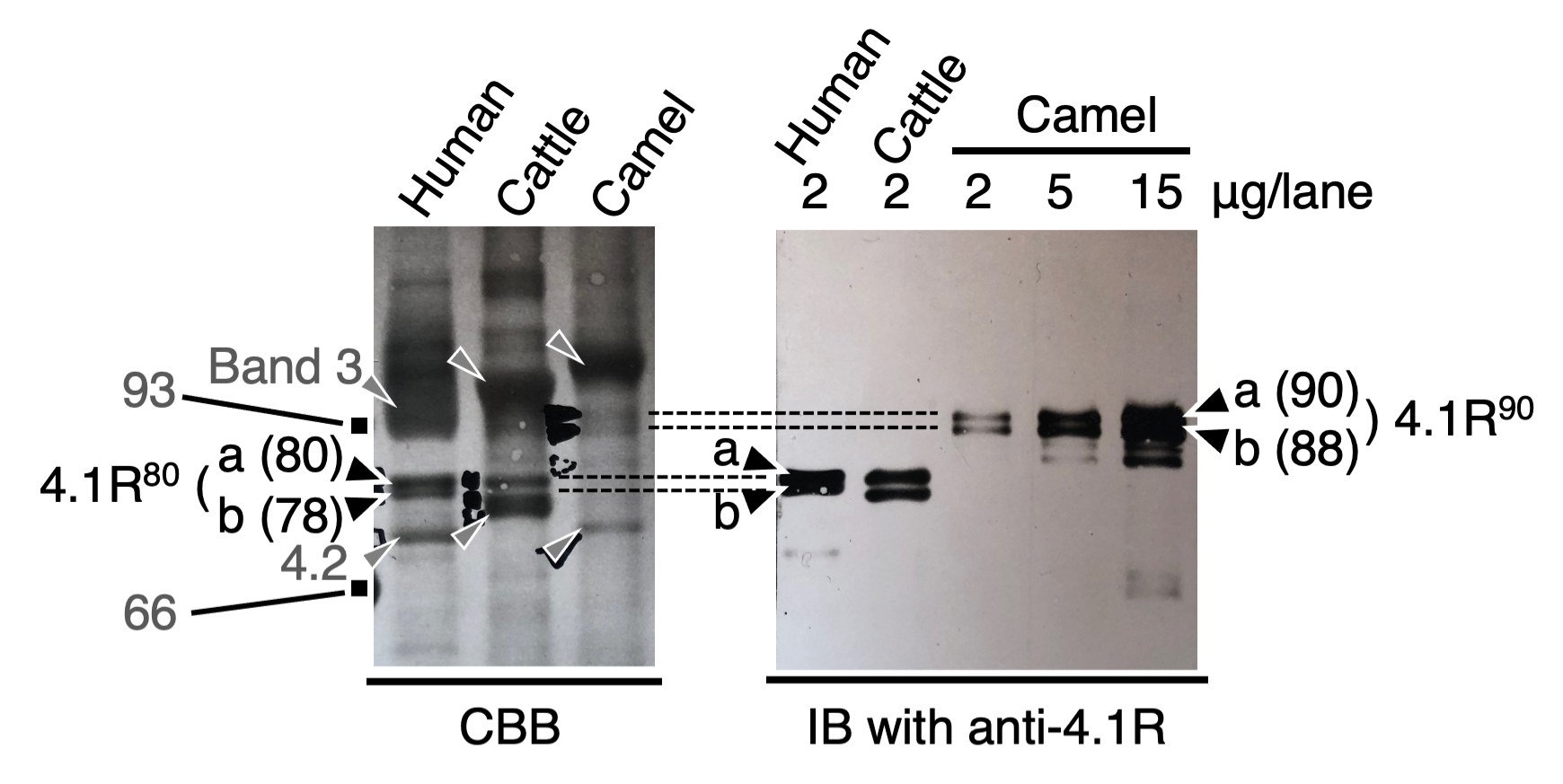


**Fig. S1. Camel RBC membranes contain a 90-kDa 4.1R larger than the highly conserved 4.1R^80^ in other mammalian RBCs.**

Membrane proteins from human, bovine, and camel RBCs were separated by SDS-PAGE on an 8% gel followed by Coomassie brilliant blue staining (*left panel,* *CBB*, 10 μg/lane) and immunoblotting using anti-canine 4.1R (*right panel, IB*, 2–10 μg/lane). The migration positions of protein 4.1R, band 3, protein 4.2, and size markers (93 kDa and 66 kDa) are indicated. The 4.1R polypeptides in human and bovine RBCs show migration positions close to each other (*4.1R^80^*, *a* and *b*), whereas the 4.1R protein in camel RBCs exhibits a slower mobility on the gel (*4.1R^90^*, *a* and *b*). SDS-PAGE and immunoblotting were performed as described previously (Inaba and Maede, 1988; Inaba *et al.*, 1996). Immunoblotting of band 3 and protein 4.2 were also performed as described (Inaba *et al.*, 1996) (data not shown).


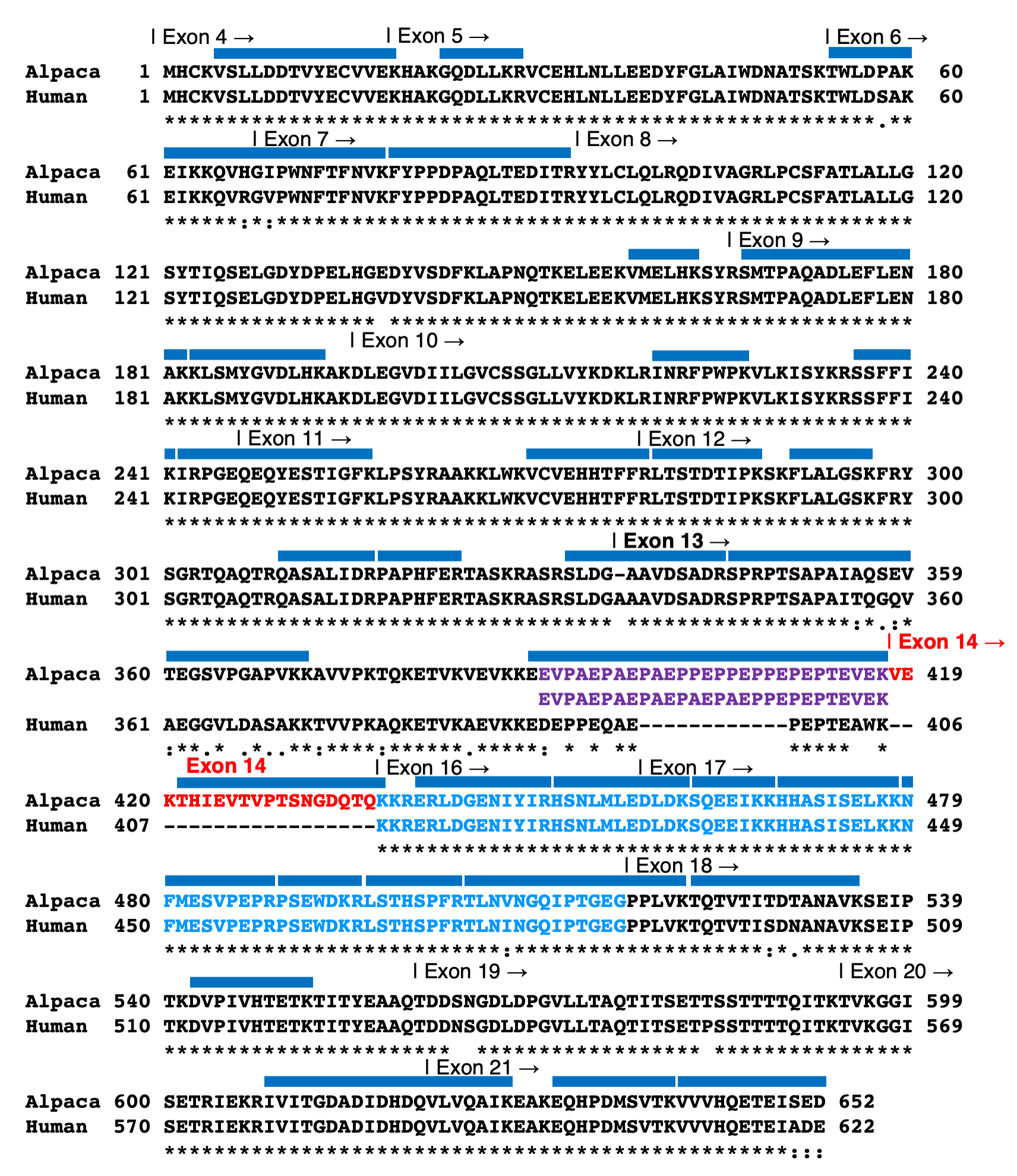


**Fig. S2. Comparison of amino acid sequences of alpaca 4.1R^90^ and human 4.1R^80^.**

Alignment of the amino acid sequences of the major forms of 4.1R in alpaca (4.1R^90^) and human (4.1R^80^) RBCs; asterisks indicate identical amino acid residues. Boundaries for exons are also shown. The alpaca 4.1R^90^ sequence was deduced from its cDNA sequence; the bars in blue above the amino acid residues indicate the peptides detected in LC-MS/MS analysis of the 4.1R^90^ polypeptide separated on SDS-PAGE as described in the text. Purple letters indicate the Pro- and Glu-rich sequence in the C-terminal region of the exon 13-derived sequence (“PE”, 28 amino acid residues); red letters indicate the exon 14-derived sequence (“e14”, 19 amino acid residues); and blue letters indicate the amino acids in the SABD. The two independent sequences in the PE region were due to allelic differences in nucleotide sequences of exon 13, one containing three PAE and three PPE repeats (GenBank accession number OM890907) and the other containing five PAE repeats and a single PPE sequence (GenBank accession number OM890908). The human 4.1R sequence is adapted from previous studies (Conboy, 1999; Inaba *et al.*, 1992; Tang *et al.*, 1988).


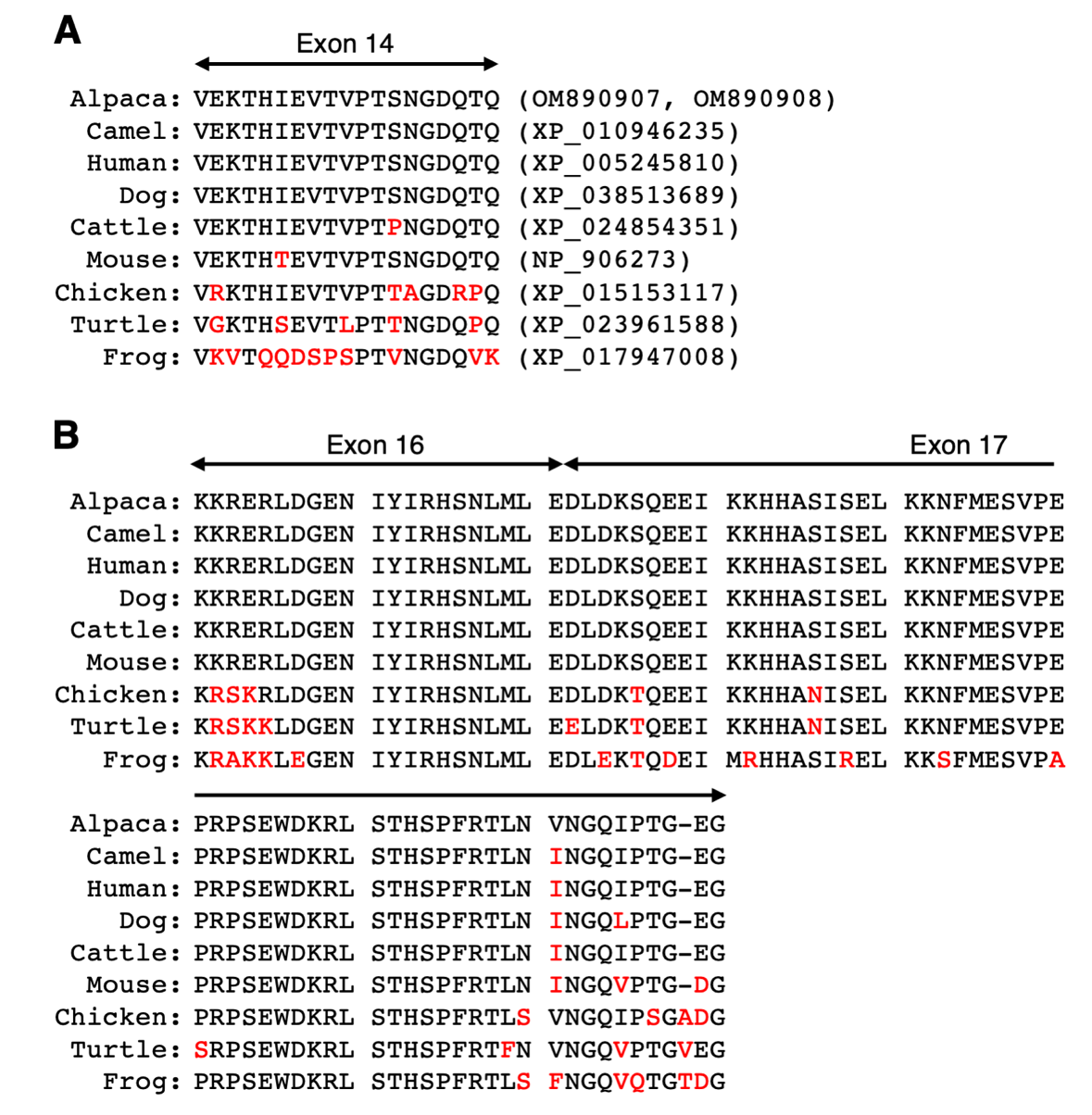


**Fig. S3. Alignment of amino acid sequences encoded by the exons 14, 16, and 17.**

Alignment of amino acid sequences of e14 (*A*, exon 14) and SABD (*B*, exons 16 and 17) in 4.1R proteins of several species. Amino acid sequences were obtained from the GenBank with accession numbers, alpaca (this study), OM890907 and OM890708; camel, XP_01946235; human, XP_005245810; dog, XP_038513689; cattle, XP_024854351; mouse, XP_906273; chicken, XP_015153117; turtle, XP_023961588; and frog, XP_01794008. Identical amino acid residues are shown in black, whereas different residues are indicated in red.

**References**

Conboy, J. (1999) The role of alternative pre-mRNA splicing in regulating the structure and function of skeletal protein 4.1. *Proc Soc Exp Biol Med* **220**, 73–78

Inaba, M., Gupta, K. C., Kuwabara, M., Takahashi, T., Benz, E. J., Jr., and Maede, Y. (1992) Deamidation of human erythrocyte protein 4.1: possible role in aging. *Blood* **79**, 3355–3361

Inaba, M., and Maede, Y. (1988) Correlation between protein 4.1a/4.1b ratio and erythrocyte life span. *Biochim Biophys Acta* **944**, 256–264

Inaba, M., Yawata, A., Koshino, I., Sato, K., Takeuchi, M., Takakuwa, Y., Manno, S., Yawata, Y., Kanzaki, A., Sakai, J., Ban, A., Ono, K., and Maede, Y. (1996) Defective anion transport and marked spherocytosis with membrane instability caused by hereditary total deficiency of red cell band 3 in cattle due to a nonsense mutation. *J Clin Invest* **97**, 1804–1817

Tang, T. K., Leto, T. L., Correas, I., Alonso, M. A., Marchesi, V. T., and Benz, E. J., Jr. (1988) Selective expression of an erythroid specific isoform of protein 4.1. *Proc Natl Acad Sci U S A* **85**, 3713–3717
